# Gastrojejunocolic fistula: a rare complication of peptic ulcer surgery

**DOI:** 10.1093/jscr/rjad125

**Published:** 2023-07-31

**Authors:** Sarra Ben Azouz, Hajer Hassine, Habiba Debbabi, Haythem Yacoub, Dhouha Cherif, Balkis Elkhouni, Seif Boukriba, Hela Kchir, Nadia Maamouri

**Affiliations:** Department of Gastroenterology B, Rabta Hospital, Tunis 8075, Tunisia; Department of Gastroenterology B, Rabta Hospital, Tunis 8075, Tunisia; Department of Gastroenterology B, Rabta Hospital, Tunis 8075, Tunisia; Department of Gastroenterology B, Rabta Hospital, Tunis 8075, Tunisia; Department of Gastroenterology B, Rabta Hospital, Tunis 8075, Tunisia; Department of Radiology, Rabta Hospital, Tunis 8075, Tunisia; Department of Radiology, Rabta Hospital, Tunis 8075, Tunisia; Department of Gastroenterology B, Rabta Hospital, Tunis 8075, Tunisia; Department of Gastroenterology B, Rabta Hospital, Tunis 8075, Tunisia

**Keywords:** gastrojejunocolic fistula, complication, peptic ulcer surgery

## Abstract

Gastrojejunocolic fistula (GJF) is a very rare complication of peptic ulcer surgery. Patients with this condition often present with diarrhea, fecal vomiting as well as weight loss. Here, we report a case of 62-year-old male with a GJF complicating upper gastrointestinal surgery.

## INTRODUCTION

Gastrojejunocolic fistula (GJF) is a very serious complication that is associated with high morbidity and mortality [1].

Post-peptic ulcer disease surgery, inadequate gastric resection or incomplete vagotomy, malignancy, tuberculosis, trauma and diverticulitis are the most causes of GJF. With the improved surgical techniques and equipment, incidence of GJF had significantly dropped and now it rarely occurs in patients with marginal or recurrent ulcer [2].

Here, we present a case of a GJF complicating upper gastrointestinal surgery.

## CASE REPORT

A 62-year-old male was diagnosed in 2020 with abdominal pain, diarrhea and eructation of fecal smelling gas. This symptomatology evolved since 6 months and was associated with a significant weight loss. He had undergone surgery for acid peptic disease in 1994. On physical examination, he had pale conjunctiva, supra umbilical surgical scar as well as lower limbs edema.

**Figure 1:**
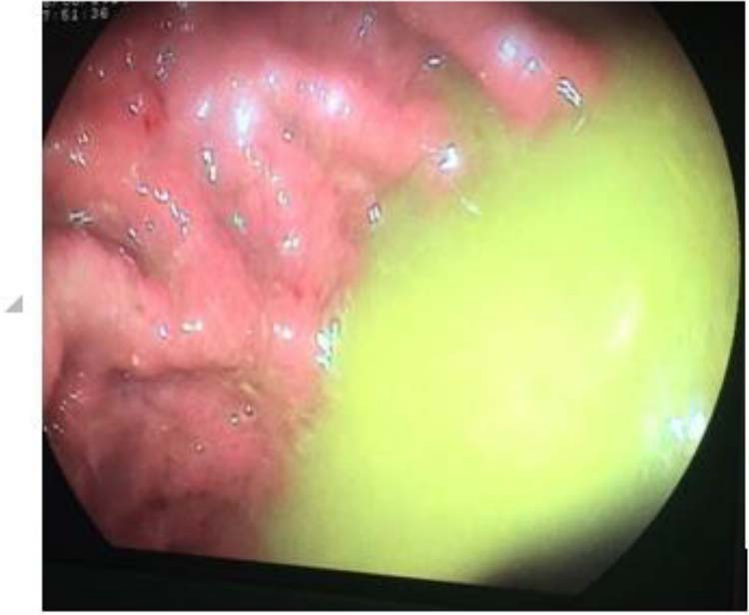
Upper endoscopy showing gastric stasis and fecal liquid in gastric cavity.

Laboratory tests found anemia (9 g/dl), hypoalbuminemia (26.1 g/dl) and hypocholesterolemia (2.6 mmol/L). Upper endoscopy showed the evidence of fecal liquid in gastric cavity (Fig. 1). Colonoscopy showed two structures with a luminal appearance at the distal transverse colon. Upon advancing the colonoscope through these structures, intestinal mucosa were identified and histopathological examination of biopsies showed jejunal and duodenal mucosa.

**Figure 2:**
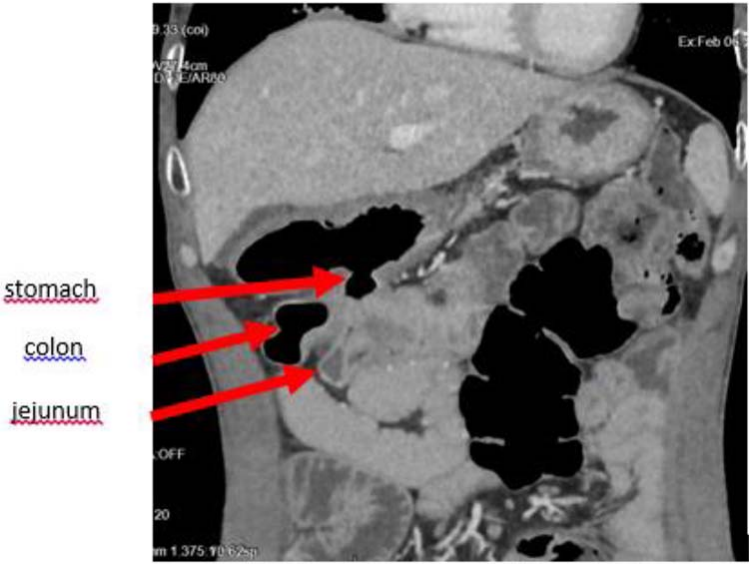
Coronal section showing the parietal defect of the greater curvature of the stomach communicating with the colon.

**Figure 3:**
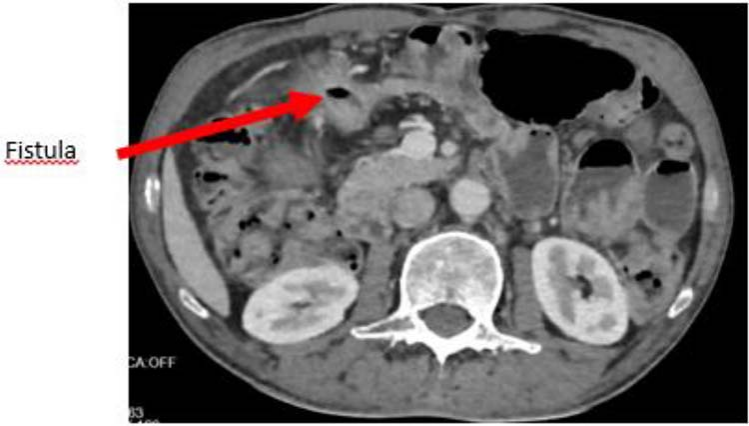
Axial section showing the large GJF with stranding of the surrounding fat.

The enteroscan showed a large fistula between the anterior face of the stomach, the jejunum and the transverse colon (Figs 2 and 3). The diagnosis of GJF was then established. The patient underwent surgery. A revision gastrectomy, truncal vagotomy and segmental resection of the jejunum and transverse colon with Roux-en-Y reconstruction were performed. During the follow-up, the patient remained well and gained weight.

## DISCUSSION

Nowadays, the necessity of surgical treatment of peptic ulcer disease has significantly decreased due to the efficacy of -roton pump inhibitors as well as the eradication regimes of *Helicobacter pylori*. Consequently, the incidence rate of GJF has been remarkably lowered because of the decrease in surgery [3].

GJF is the late complication of inappropriate surgery resulting from simple gastroenterostomy, inadequate gastric resection or incomplete vagotomy. It could appear in 20 years or more after surgery [4].

Classically, GJF present with a triad of diarrhea, feculent vomiting and weight loss. However, this triad is seen in only 30% of cases [5]. Our patient was diagnosed with these three symptoms.

Diarrhea and weight loss are present in 80% of cases. Eructation of fecal-smelling gas or fecal vomiting are marked features for diagnosis and often cause severe embarrassment for patients. Other signs such as a loss of appetite, abdominal pain and edema could be identified, like in our case [2].

Diagnosis of this disease is based on showing the passage of the stomach or jejunum into the transverse colon [2, 6].

Barium enema, which was the usual diagnostic test for GJF, has been replaced by tomography computers today. In the current case, the diagnosis was made through endoscopy and tomography computers. The diagnosis can also be made on histological identification of jejunal mucosa in the fistula like in this case. There is no need for further confirmatory investigations [4, 7].

The conventional treatment for GJF involves improving the nutritional status and two-to-three-phased operations with colostomy to minimise mortality and morbidity. Today, however, due to improved parenteral and enteral support treatment, single-stage procedures can be applied and these have been favored to minimise mortality [4, 8].

With regard to definitive surgery, simple repair is associated with a high recurrence rate, as the predisposing factors for gastric ulcer still remain in place. If a gastric remnant remains, a revision gastrectomy to remove excessive antrum should be done. If vagotomy has not previously been completed, it should be performed. Recurrence is rare with the above definitive surgery [9, 10]. Our patient became asymptomatic and gained weight during the follow-up.

## CONCLUSION

GJF is rare and is mainly developed as a result of inadequate resection of the stomach or incomplete vagotomy in the past. Clinical suspicion should be done in the presence of chronic diarrhea, fecal vomiting, abdominal pain and features of malnutrition in patients with a history of peptic ulcer surgery. The diagnosis is possible using upper gastrointestinal endoscopy and tomography computers. The modern management of GJF includes improving the metabolic status as well as definitive surgery with revision gastrectomy and/or truncal vagotomy.

## AUTHORS’ CONTRIBUTIONS

Sarra Ben Azouz: concept, design, definition of intellectual content, literature search, manuscript preparation, manuscript review.

Hajer Hassine: concept, design, definition of intellectual content, manuscript preparation, manuscript editing, manuscript review.

Habiba Debbabi: definition of intellectual content, manuscript preparation, manuscript review.

Haythem Yacoub: design, manuscript review.

Dhouha Cherif: design, manuscript review.

Balkis Elkhouni: concept, design, definition of intellectual content, manuscript preparation.

Boukriba Seif: Design, manuscript review.

Héla Kchrir: Design, manuscript review.

Nadia Maamouri: Definition of intellectual content, manuscript review.

## GUARANTOR

Sarra Ben Azouz.

## CONFLICT OF INTEREST STATEMENT

None declared.

## FUNDING

None.

## PATIENT CONSENT

Patient gave an informed consent.

## DATA AVAILABILITY

No data are associated with this article.
